# Examining rehabilitation access disparities: an integrated analysis of electronic health record data and population characteristics through bivariate choropleth mapping

**DOI:** 10.1186/s12913-024-10649-1

**Published:** 2024-02-07

**Authors:** Sang S. Pak, Madeline Ratoza, Victor Cheuy

**Affiliations:** 1 Department of Physical Therapy and Rehabilitation Science, School of Medicine, University of California San Francisco, 1500 Owens St Suite 400, San Francisco, CA 94158 USA; 2 College of Rehabilitative Sciences, University of St. Augustine for Health Sciences, Austin, TX USA; 3 Department of Radiology and Biomedical Imaging, University of California San Francisco, San Francisco, CA USA

**Keywords:** Patient access, Rehabilitation, Geographic information systems, Health disparity, Bivariate choropleth maps, Electronic health records

## Abstract

**Background:**

Despite efforts to view electronic health records (EHR) data through an equity lens, crucial contextual information regarding patients’ social environments remains limited. Integrating EHR data and Geographic Information Systems (GIS) technology can give deeper insights into the relationships between patients’ social environments, health outcomes, and geographic factors. This study aims to identify regions with the fastest and slowest access to outpatient physical therapy services using bivariate choropleth maps to provide contextual insights that may contribute to health disparity in access.

**Methods:**

This was a retrospective cohort study of patients’ access timelines for the first visit to outpatient physical therapy services (*n* = 10,363). The three timelines evaluated were (1) referral-to-scheduled appointment time, (2) scheduled appointment to first visit time, and (3) referral to first visit time. Hot and coldspot analyses (CI 95%) determined the fastest and slowest access times with patient-level characteristics and bivariate choropleth maps that were developed to visualize associations between access patterns and disadvantaged areas using Area Deprivation Index scores. Data were collected between January 1, 2016 and January 1, 2020. EHR data were geocoded via GIS technology to calculate geospatial statistics (*G*_*i*_^*∗*^ statistic from ArcGIS Pro) in an urban area.

**Results:**

Statistically significant differences were found for all three access timelines between coldspot (i.e., fast access group) and hotspot (i.e., slow access group) comparisons (*p* < .05). The hotspot regions had higher deprivation scores; higher proportions of residents who were older, privately insured, female, lived further from clinics; and a higher proportion of Black patients with orthopaedic diagnoses compared to the coldspot regions.

**Conclusions:**

Our study identified and described local areas with higher densities of patients that experienced longer access times to outpatient physical therapy services. Integration of EHR and GIS data is a more robust method to identify health disparities in access to care. With this approach, we can better understand the intricate interplay between social, economic, and environmental factors contributing to health disparities in access to care.

**Supplementary Information:**

The online version contains supplementary material available at 10.1186/s12913-024-10649-1.

## Introduction

Social and structural determinants of health are the underlying contributing factors to health disparities [1]. One important domain of social determinants of health is healthcare access [2]. Access is a complex metric, but often defined as “the timely use of personal health services to achieve the best health outcomes” [3]. Access to healthcare is a fundamental human right for people of all races, ethnicities, and sociopolitical-economic status [4]. While the Affordable Care Act (ACA) increased insurance coverage significantly and lowered the uninsured rate to a record low in 2021, access to care involves more than just having insurance [5]. For example, in the 2022 National Healthcare Quality and Disparities Report, a significant disparity was observed among insured, non-White adults who experienced extended wait times for specialist appointments within the past year [6]. Moreover, investigations into health disparities within primary care clinics and specialized practices have indicated that additional social determinants like education, cultural norms, transportation needs, and financial costs influence healthcare access inequities [7–9].

With the prevalent use of Electronic Health Record (EHR) data in ambulatory settings [10], opportunities are available to identify, monitor, and explore inequities in care and investigate their contributing factors [11]. However, despite our best efforts to view EHR data through an equity lens [11], crucial contextual information regarding patients’ social environments remains limited [12–14]. To address these contextual concerns, an emerging approach is to link other publicly available socioeconomic status (SES) data to patients’ EHR data [15]. Specifically, Geographical Information Systems (GIS) offer a promising avenue for robust spatial analyses of health outcomes at the contextual-level [16]. Further, the capacity to unveil spatial relationships between paired thematic variables such as SES data and health outcomes through bivariate choropleth maps holds the potential to illuminate new findings [17]. Integrating SES data and GIS technology with EHR data can give deeper insights into the relationships between patients’ social environments, health outcomes, and geographic factors [18–20]. To date, there has been minimal research on sociodemographic disparities related to access timelines related to outpatient physical therapy (PT) services using EHR and GIS data sources.

The purpose of this study is to investigate groups of individuals who initiate care quickly compared to those that initiate care slowly by describing the sociodemographic and neighborhood characteristics of where each group of patients reside. The behavioral ecological model of healthcare access supports this framework by describing how an individual exists within an environment comprising social, healthcare, neighborhood, and built environments [21]. These environments and individual characteristics influence health behaviors and realized access to care, which in turn impact health outcomes [21]. This study has three main aims. Primary to use timelines composed of three access metrics from the electronic health record to geospatially determine individuals who initiate care very quickly (coldspot) compared to those who take the longest (hotspot) to initiate care. Second, to evaluate sociodemographic and neighborhood characteristics of the hot and coldspot regions to investigate factors that may be associated with disparities of access patterns. Finally, to employ bivariate choropleth maps to visualize access metrics and neighborhood-level disadvantage using Area Deprivation Index (ADI) Scores. The neighborhood characteristics provide additional context to the factors that may influence the care delay.

## Methods

### Study population

The study cohort included San Francisco County residents over 18 years of age who had received outpatient physical therapy services between the dates of January 1, 2016 and January 1, 2020 at a large urban academic medical center. Patients with all conditions with a referral to an outpatient setting were included in the study. Any residents outside of San Francisco County were excluded from the study. This study protocol was approved by the University of California San Francisco Institutional Review Board (19–28,255).

### Data sources

An EHR dataset was extracted from the institution’s clinical data warehouse (CDW) consisting of patient-level data: age, sex, race (White, Asian, Black, Other, Unknown), ethnicity (Hispanic, non-Hispanic, Unknown), insurance payer type (Private, Medicaid, Medicare, Other), Area Deprivation Index (ADI), and distance (miles) from home to clinic. The dataset also includes geocoded data of latitude and longitude coordinates for patient addresses, census tract IDs, and block group IDs prepared by the UCSF Population Health Data Initiative (PHDI) team [22, 23]. By using census block group ID number assigned to individual patient record, we were able also to link it to neighborhood-level data. From the patient-level data, three access timelines were evaluated. They included: 1) referral-to-schedule time, defined as the time elapsed between when patients receive a referral to physical therapy and when patients initiate care, 2) schedule-to-appointment time, defined as the time that elapses between when a patient calls the clinic to schedule and when they arrive for their first visit, and 3) referral-to-appointment time defined as the total time from referral to first completed appointment. Other utilization metrics included the number of visits, cancellations (total and number of same-day cancellations), and diagnosis type (orthopedic, neurologic, pelvic, other).

### Area deprivation index

The Area Deprivation Index (ADI) is a multi-faceted proxy measure derived from several different variables, including education level, employment, income, housing quality, and access to services [24]. A higher score indicates a greater level of socioeconomic disadvantage [24] and can be derived for both a National and State-specific level for California [25]. Our analysis used State-specific rather than National scores to be locally sensitive and accurately capture the region’s local comparability [26]. Using Spatial Join in ArcGIS Pro (2.8.3) [27], the census block group ID numbers from patient geocoded data were linked with the ADI scores (ADI, 2020 dataset) to understand and visualize the potential socioeconomic disadvantage of geographical regions of patients’ residences. We mapped the geocoded patients with ADI scores [24, 25].

### Geospatial analysis: inquiry into residential areas of patients with the longest (hotspot) and shortest (coldspot) access metric times

**Fig. 1:**
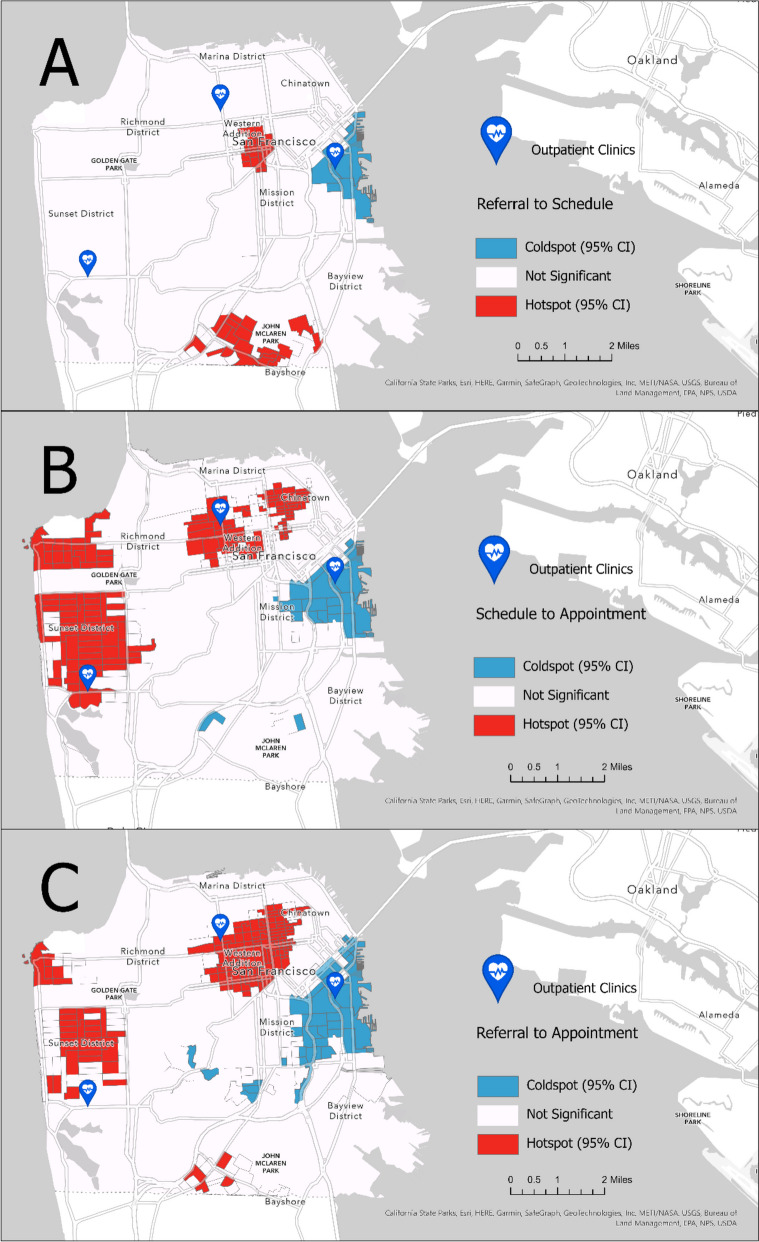
*Hotspots (red) and coldspots (blue) for the access timelines* (Map **A**: referral-to-schedule, Map **B**: schedule-to-appointment, Map **C**: referral-to-appointment)

Similar methodology as described by Kethireedy et al. [28] was used to calculate geospatial statistics for patients’ time-based access metrics and ADI state-level scores [24]. By using individual geocoded patient data prepared by the UCSF Population Health Data Initiative (PHDI) team [22, 23], we used individual patient records that contained three time-based metrics aggregated to census block group level, we aggregated to the census block group. Using Getis–Ord *G*_*i*_^*∗*^statistic feature from ArcGIS Pro (version 2.8.3) [29, 30], geospatial clustering analysis of three distinct time-based metrics (i.e., referral-to-scheduled time, scheduled-to-appointment time, and referral-to-appointment time) was assessed to identify patterns and groupings in spatial data. Several steps were taken to calculate the required input parameters for *G*_*i*_^*∗*^statistics, and they are described in the supplementary file (See Supplemental File). A statistically significant “hot spot” represents a higher-value feature surrounded by other higher-value neighboring features. Conversely, a statistically significant “coldspot” refers to a lower-value feature surrounded by other lower-value neighboring features [29]. *G*_*i*_^*∗*^ statistic contain a z-score [31] and clusters with a 95% significance level from a two-tailed normal distribution. A z-score close to zero and a *p*-value greater than 0.05 suggest complete spatial randomness within the study area. On the other hand, a positive z-score and a *p*-value less than 0.05 signify the clustering of high values. The hot and coldspot analysis results were calculated with a 95% CI (Fig. 1). Lastly, clusters were compared using the False Discovery Rate correction in the analysis to account for potential false positive hot spots [32].

### Secondary measures: neighborhood characteristics

To understand neighborhood-level characteristics of the regions in the hot and coldspot analysis, several data sources were used, including CDC PLACES data, the American Community Survey, California Healthy Places index from Public Health Alliance. Neighborhood-level characteristic data are summarized in Table 1.

**Table 1:** Neighborhood-level characteristics

**Prevalence Measures**	
Disability	Percent of people of all ages living within a census tract who have disability [33]
Arthritis	Percent of adults age 18 and over who have been told by a healthcare provider they have arthritis within a census tract or ZIP Code Tabulation Area [34]
Obesity	Percent of adults with a BMI of at least 30 kg/m2 [34]
Mental Health	Poor mental health is reported as the percent of adults age 18 and over living within a census tract or ZIP Code Tabulation Area who reported, at the time of the survey, their mental health was not good for 14 days or more of the past 30 days [34]
Physical Health	Physical health is reported as the percent of adults age 18 and over living within a census tract or ZIP Code Tabulation Area who reported, at the time of survey, their physical health was not good for 14 days or more of the past 30 days [34]
Physical inactivity	Percent of adults who answered “no” to the question, “During the past month, other than your regular job, did you participate in any physical activities or exercises such as running, calisthenics, golf, gardening, or walking for exercise?” [34]
**Neighborhood Indices**	
Healthy Places Index	Combines 25 community characteristics that predict life expectancy and influence health [35]

### Bivariate choropleth: hotspots and coldspots with ADI scores

**Fig. 2:**
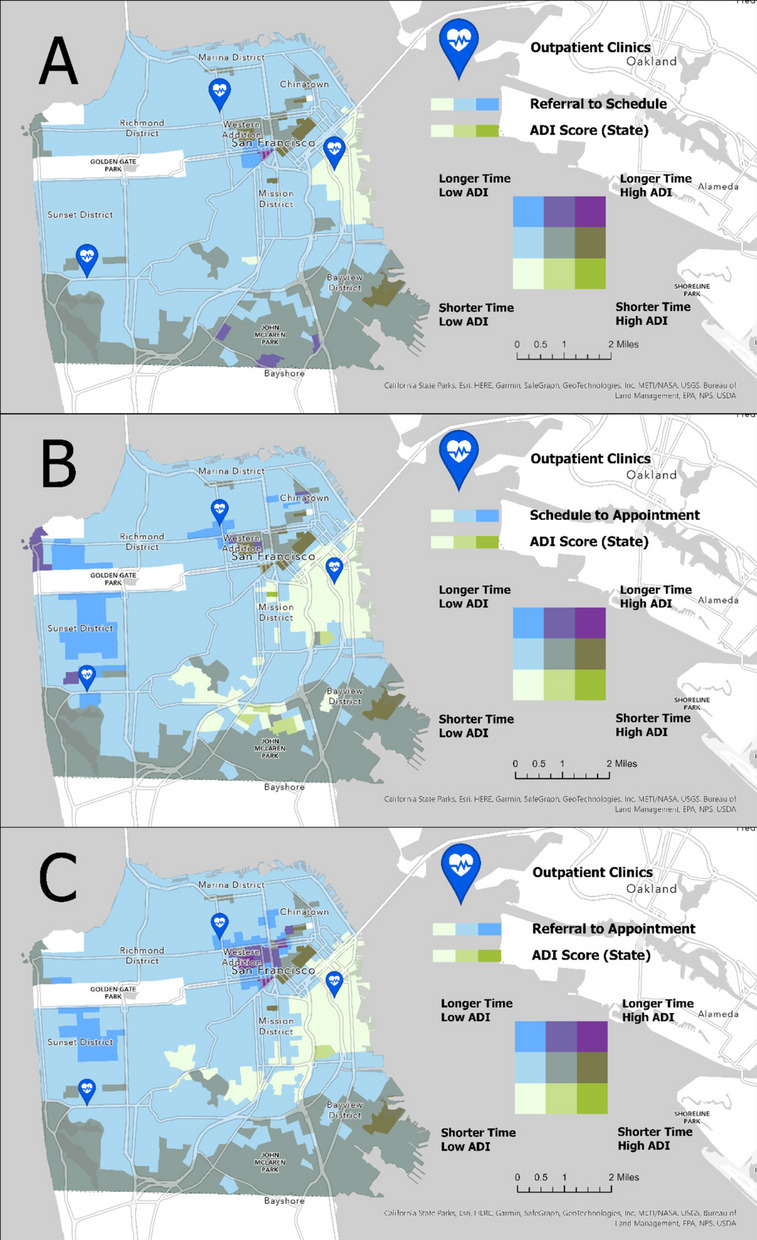
*Bivariate choropleth maps of timelines and ADI scores* (Map **A**: referral-to-schedule, Map **B**: schedule-to-appointment, Map **C**: referral-to-appointment)

A bivariate choropleth map was created to visually describe relationships between two distinct variable classes (clusters of time-based access metrics resulting in hot and coldspots and ADI State scores) on a projected layer. Each variable (i.e., access metric and ADI score) was assigned a different graded color scheme to delineate relationships of high and low respective values within a 3 × 3 table (Fig. 2). While there are different classification approaches for bivariate choropleth maps, we applied equal intervals to classify groupings with the same hot and coldspots with 95% CI and ADI scores [36, 37], and to highlight changes in the extreme points with a relatively intuitive view for readers [36, 38]. For example, Fig. 2 (Map A) highlights the “Longer” and “Shorter Times” of the referral to schedule metric with an overlay of graded colors of ADI scores reflecting least to most disadvantaged block groups labeled as “High” and “Low” ADI in shades of color green. Similarly, Figs. 2 (Map B & C) show longer and shorter times of the schedule to appointment metric and referral to the first appointment metrics with ADI scores, respectively.

### Statistical analysis

Medians and interquartile ranges (IQR) were calculated for all continuous variables due to the non-normality of the data as determined by the Kolmogorov–Smirnov Test. Hot and coldspot regions and neighborhood characteristics were compared using Mann–Whitney U tests for continuous variables and Chi-square tests for all nominal data, with a significance level of *α* = .05. SPSS Statistics V27 (IBM, USA) was used for all analyses.

## Results

### Demographics

The data queried yielded 10,363 unique patient records after removing missing or erroneous data pertaining to our primary outcome measuresThe cohort had a median (IQR) age of 54 (40, 67) years; it was 63% female, and 53% non-Hispanic White (Table 2). The payer type was predominately Private (42%), and the referral type was predominately for orthopedic diagnoses (69%). Median access times for care were 3 (0, 10), 9 ( 5, 15), and 15 (9, 26) days for referral-to-scheduled time, scheduled-to-appointment time, and referral-to-appointment time, respectively.

**Table 2:** Total sample summary (*N* = 10,363)

Variable	Median (IQR) or N (%)
Age (years)	54 (40, 67)
Sex	
*Female*	6536 (63)
Race	
*White*	5485 (53)
*Asian*	2657 (26)
*Black*	740 (7)
*Other*	1384 (13)
*Unknown*	97 (1)
Ethnicity	
*Hispanic*	1025 (10)
*Unknown*	229 (2)
Payer	
*Private*	4310 (42)
*Medicaid*	1808 (17)
*Medicare*	2652 (26)
*Other*	1593 (15)
State ADI	1 (1, 2)
Diagnosis	
*Orthopaedic*	7147 (69)
*Neurologic*	946 (9)
*Pelvic*	738 (7)
*Other*	1532 (15)
Time (days)	
*Referral to Scheduled*	3 (0, 10)
*Scheduled to Appointment*	9 (5, 15)
*Referral to Appointment*	15 (9, 26)
Time (% of Ref to Appt)	
*Referral to Schedule*	26 (0, 57)
*Scheduled to Appointment*	73 (42, 100)
Visits	4 (2, 7)
Cancellations	
*Total*	2 (1, 4)
*Same day*	0 (0, 1)
Clinic distance (miles)	2.40 (1.44, 3.61)

*CCI* Charlson Comorbidity Index, *ADI* Area Deprivation Index, scores range from 0 (least deprived) to 10 (most deprived)

### Geospatial analyses

For referral-to-scheduled time (Fig. 1, Map A), between-group statistics found that the hotspot (i.e., longer time) cohort was older (51 vs 44 years) lived farther from the clinic (2.03 vs 1.42miles), and had a higher proportion of Black patients (25% vs 5%) and patients with Medicaid (25% vs 11%) and Medicare (28% vs 17%) (Table 3). For scheduled-to-appointment time (Fig. 1, Map B), the hot spot cohort was older (56 vs 46 years), lived farther from the clinic (2.59 vs 1.61 miles), and had higher proportions of Females (65% vs 59%), Asians (41% vs 25%), non-Hispanic patients (92% vs 88%), Medicare (29% vs 16%) and Other payer types (18% vs 9%), and neurologic-related diagnoses (9% vs 5%) (Table 3). For referral-to-appointment time (Fig. 1, Map C), the hot spot cohort was older (55 vs 47 years), lived farther from the clinic (2.07 vs 1.71 miles), lived in locations of higher ADI scores (2 vs 1), and had higher proportions of Asian (28% vs 23%), Black (15% vs 4%), and non-Hispanic patients (90% vs 88%), and Medicaid (23% vs 13%) and Medicare (33% vs 17%) payer types (Table 3).

**Table 3:** Access time analyses

Variable	*Referral to Scheduled Time*			*Scheduled to Appointment Time*			*Referral to Appointment Time*		
	Coldspot	Hotspot	*P*-value	Coldspot	Hotspot	*P*-value	Coldspot	Hotspot	*P*-value
Age (years)	44 (34, 59)	51 (36, 64)	.018	46 (34, 58)	56 (42, 69)	< .001	47 (36, 61)	55 (40, 68)	< .001
Sex			.508			.024			.290
*Female*	222 (57)	133 (59)		374 (59)	517 (65)		546 (60)	576 (62)	
Race			< .001			< .001			< .001
*White*	214 (55)	90 (40)		355 (56)	354 (44)		532 (59)	412 (45)	
*Asian*	107 (27)	41 (18)		160 (25)	325 (41)		208 (23)	257 (28)	
*Black*	19 (5)	55 (25)		28 (4)	38 (5)		38 (4)	134 (15)	
*Other*	46 (12)	38 (17)		83 (13)	79 (10)		119 (13)	114 (12)	
*Unknown*	6 (2)	0 (0)		10 (2)	4 (1)		12 (1)	5 (1)	
Ethnicity			.251			.014			.027
*Hispanic*	32 (8)	26 (12)		58 (9)	56 (7)		84 (9)	73 (8)	
*Unknown*	12 (3)	4 (2)		21 (3)	11 (1)		31 (3)	15 (2)	
Payer			< .001			< .001			< .001
*Private*	248 (63)	79 (35)		403 (63)	303 (38)		545 (60)	301 (33)	
*Medicaid*	43 (11)	57 (25)		78 (12)	124 (16)		120 (13)	215 (23)	
*Medicare*	66 (17)	62 (28)		100 (16)	229 (29)		155 (17)	304 (33)	
*Other*	35 (9)	26 (12)		55 (9)	144 (18)		89 (10)	102 (11)	
State ADI	2 (1, 2)	2 (1, 3)	.001	1 (1, 2)	1 (1, 2)	.887	1 (1, 2)	2 (1, 3)	< .001
Diagnosis			.057			.013			.254
*Orthopaedic*	290 (74)	152 (68)		461 (72)	559 (70)		646 (71)	653 (71)	
*Neurologic*	17 (4)	20 (9)		30 (5)	73 (9)		55 (6)	72 (8)	
*Pelvic*	30 (8)	13 (6)		53 (8)	57 (7)		81 (9)	65 (7)	
*Other*	55 (14)	39 (17)		92 (14)	111 (14)		127 (14)	132 (14)	
Time (days)									
*Referral to Scheduled*	1 (0, 7)	4 (1, 13.75)	< .001	2 (0, 7)	1 (3, 9)	< .001	2 (0, 8)	4 (1, 11)	< .001
*Scheduled to Appointment*	8 (4, 13)	9 (5, 15)	.023	8 (4, 13)	10 (6, 17)	< .001	8 (4, 13)	9 (5.75, 16)	< .001
*Referral to Appointment*	11.5 (6, 21)	15 (9, 30)	< .001	12 (6, 22)	17 (10, 26)	< .001	12 (7, 22)	16 (9, 28)	< .001
Time (% of Ref to Appt)									
*Referral to Schedule*	12 (0, 47)	31 (7, 64)	< .001	16 (0, 50)	25 (5, 53)	< .001	18 (0, 50)	28 (5, 59)	< .001
*Scheduled to Appointment*	86 (50, 100)	69 (36, 93)	< .001	83 (50, 100)	75 (46, 95)	.001	80 (45, 100)	71 (41, 94)	< .001
Visits	4 (3, 7)	4 (2, 6)	.120	4 (2, 7)	4 (2, 7)	.122	4 (2, 7)	4 (2, 7)	.185
Cancellations									
*Total*	2 (1, 3)	2 (1, 3)	.758	2 (1, 3)	2 (1, 3)	.542	2 (1, 3)	2 (1, 3)	.592
*Same day*	0 (0, 1)	0 (0, 1)	.180	0 (0, 1)	0 (0, 0)	.025	0 (0, 1)	0 (0, 1)	.963
Clinic distance (miles)	1.42 (0.47, 2.78)	2.03 (1.31, 3.62)	< .001	1.61 (066, 2.80)	2.59 (1.41, 3.91)	< .001	1.71 (0.77, 2.95)	2.07 (1.27, 3.45)	< .001

Data presented as Median (IQR) or N (%)

*CCI* Charlson Comorbidity Index

### Neighborhood characteristics

Neighborhood characteristics of the hot and coldspots for each access metric are summarized in Table 4. Consistently higher proportions of disability, arthritis, and self-reported poor physical health and physical inactivity were found for all access metrics for hot spot neighborhoods (*p* < .001). Lower Healthy Places Indices were also found in the referral-to-schedule and referral-to-appointment times (*p* < .001). Proportions of obesity and reported poor mental health had inconsistent results, being higher in the hot spot neighborhood of the referral-to-scheduled time (*p* < .001) but lower in the scheduled-to-appointment time (*p* < .001).

**Table 4:** Neighborhood characteristic analysis

Variable	*Referral to Scheduled Time*			*Scheduled to Appointment Time*			*Referral to Appointment Time*		
	Coldspot Neighborhood	Hotspot Neighborhood	*P*-value	Coldspot Neighborhood	Hotspot Neighborhood	*P*-value	Coldspot Neighborhood	Hotspot Neighborhood	*P*-value
Total population (n)	52,903	96,708		151,758	223,656		135,968	240,366	
Disability (n (%))	3660 (7)	10,129 (10)	< .001	14,144 (9)	24,124 (11)	< .001	11,250 (8)	28,311 (12)	< .001
Arthritis (n (%))	6530 (12)	15,952 (17)	< .001	21,991 (15)	39,642 (18)	< .001	19,815 (15)	39,649 (16)	< .001
Obesity (n (%))	8920 (17)	17,597 (18)	< .001	27,553 (18)	33,747 (15)	< .001	24,679 (18)	39,878 (17)	< .001
Poor mental health (n (%))	5302 (10)	10,950 (11)	< .001	16,429 (11)	22,962 (10)	< .001	14,196 (10)	25,990 (11)	< .001
Poor physical health (n (%))	3824 (7)	10,074 (10)	< .001	13,734 (9)	21,707 (10)	< .001	11,363 (8)	22,945 (10)	< .001
Physical inactivity (n (%))	6768 (13)	19,607 (20)	< .001	25,778 (17)	41,425 (19)	< .001	19,948 (15)	42,885 (18)	< .001
HPI (median (IQR))	95 (92, 99)	74 (60, 85)	.001	81 (66, 93)	81 (74, 93)	.892	93 (78, 97)	78 (64, 91)	.004

*HPI* Healthy Places index

### Bivariate choropleth maps of referral to access metrics and ADI scores

Three bivariate choropleth maps (Fig. 2 Maps A-C) visualize the relationships between each access measure’s hot and coldspots and ADI Scores at the census block group, where each polygon was shaded with a unique color combination representing a distribution of high and low values from each feature variable of interest. The top bivariate map (Fig. 2, Map A) shows residential areas at census block group in San Francisco where a longer referral-to-schedule time and higher ADI scores diverge in spatial relationships – with the longest access time and highest ADI in dark purple and other combinations in varying colors.

## Discussion

### Insights from integrated patient-level and neighborhood-level data

Our methods and study results may inform administrators and policymakers of disparities contributing to variability in time-based access metrics in an urban outpatient physical therapy setting. By merging individual patient data with neighborhood characteristics linked to social determinants of health at the census block group [24], we gain insights into associations between disadvantaged communities and patients experiencing extended access times. Our analysis in San Francisco revealed a cluster of patients with prolonged access times in regions exhibiting neighborhood attributes with higher disability rates [33], lower mental and physical well-being [34], and lower socioeconomic status [24, 39] (Table 4). These findings align with a systematic review by Dawkins et al., who found that in high-income countries like the US, patients with more severe physical and mental comorbidities had more limited access to healthcare [40]. Further, our analysis from hot and coldspots juxtaposes the two extreme ends of access patterns consistent with prior research by Gao et al., who found limited access to rehabilitation services in areas of higher disability prevalence where potential demands are most needed [20].

Considering that as much as 50 percent of health outcomes are affected by social, economic, and environmental factors [41], it’s unsurprising that such profound discrepancies in a delay of care are shown in our study. Various data sources from health equity tools like the Healthy Places Index arose from the need to establish objective index score rankings to target interventions towards the most pressing needs of policymakers [35, 39], supporting integration of neighborhood-level and patient datasets to continue the efforts to combat health disparities.

Delineating the timelines of three access metrics captures important distinctions of provisioning care between the patient and the scheduler. For example, the referral-to-schedule measure depends on patients initiating care after providers create the referral. Conversely, the schedule-to-appointment measure is influenced by both patients and clinics because scheduling teams need to find a time that accommodates both availability of patients and providers. Based on the hot spot patterns from the three distinct measures, we were able to quantify the extreme differences in the timing of access patterns starting with the time to schedule by patients. Operationally, the referral-to-schedule measure showed a median value of four business days for the hot spot group and only one day for the coldspot group (*P* < .001). Variance of four business days may seem minor, but there is significant difference between hot and coldspot groups with respect to the proportion of time devoted to scheduling relative to the overall time taken: 31% to 12%, respectively, as indicated in Table 3 (*P*< .001). Moreover, any additional delay in timely care could result in adverse effects on pain, quality of life and psychological symptoms for patients waiting for physical therapy services [42].

While the urgency to schedule may also depend on patients’ personal values [43], recent studies highlight several barriers to access, particularly those from marginalized groups: navigating complex health systems to specialty care, financial burdens associated with gaps in insurance coverage, loss of time from work, and travel costs [40, 44–46]. Further research is needed to fully identify the need of our local neighborhood to deliver interventions that acknowledge their values for rehabilitation services. Nevertheless, our findings raise awareness of health disparities in care access, which warrants discussions with healthcare leaders and policymakers to bridge these gaps.

### Bivariate map visualization of access metrics and ADI scores

Combining two distinct variables from patient and neighborhood-level data – access metrics and ADI scores, respectively – can be helpful to visually provide spatial patterns of different access times with neighborhood characteristics. The gradient colors in the 3 × 3 table (Fig. 2, Maps A-C) reflect the range values from each and the combination of two variables. For example, our bivariate map identifies areas with hot spot regions (i.e., longest access time) that also spatially overlap with areas with higher ADI scores at the census group block. In Fig. 2 (Maps A-C), the purple colors represent the patient resident location with the longest access time and the highest ADI scores. While the median ADI values between hot and cold spot regions may not seem significantly different, the interquartile range values are more positively skewed for hot spot regions (Table 3). Further, other social determinants of health variables such as income, employment, and housing quality may be contributing factors. Numerous studies suggest that socioeconomic neighborhood disadvantage, as expressed through ADI scores, can predict health outcomes such as hospital readmission rates [47], observation stays [48], and mortality rates [49]. ADI scores could serve as a valuable screening tool to inform clinicians and health systems to proactively engage patients returning from their challenging environments [26]. Moreover, we can investigate the degree of neighborhood disadvantages potentially contributing to differences in health utilization using visuals of these extreme ends of access patterns. Such additional neighborhood characteristic data provides further context to the factors that may influence the care delay [50].

#### A synergy of EHR data and GIS

Using geocoded EHR data allows for direct knowledge of access information for our local population and to perform spatial analysis. The SES variables for EHR data used in our study deepened our understanding of access patterns, which is useful for resource planning based on neighborhood needs and geographic and health utilization characteristics [51, 52]. Integrating neighborhood-level data from public data sources enabled an understanding of patients’ context and the potential external barriers to access, employing an opportunity to make decisions to deliver care with “an eye on spatial equity” [18].

#### Limitations

There are several limitations to our study. First, our primary data is from one large academic health center in an urban area, and the results may not be generalizable to other areas. While the access metrics gathered in the study had three distinct time points that reflected the referral workflow, this process may not apply to all health systems or ambulatory settings. Secondly, this cross-sectional retrospective data may not always provide future access patterns or utilization due to multiple external factors such as supply, demand, and distance decay [53]. Therefore, one should be cautious about using this information in predictive planning. Thirdly, we must be cautious about not making potential pitfalls of ecological fallacy, which is making inferences about specific groups of individuals solely based on neighborhood-level characteristics [54]. Further, this study did not contain standardized patient-reported outcome measures that may have provided further information about patients’ progress in their care despite having different access patterns. However, we believe other institutions can replicate this study for their population, especially with respect to measuring ongoing access to care, which is critical to health equity work.

#### Future direction

There are several future directions from this line of research. The EHR data was collected before the COVID-19 pandemic from January 2015 to January 2020. Given the subsequent widespread adoption of telehealth, studying access pattern changes during and post-pandemic could offer valuable insights into how telehealth can contribute to equity. This is particularly relevant to this region as telehealth has been implemented in outpatient rehabilitation [55, 56]. Additionally, it will be important to understand how technical-digital barriers, such as lack of internet broadband access, affect disparity in access to telehealth services. Lastly, future research can leverage direct patient-level EHR data, including self-reported quality of life and patient-reported outcomes, to investigate how these may vary with patient neighborhood demographics.

## Conclusion

Studying access patterns remains vital to undertaking health equity work. Leveraging the combined use of Electronic Health Record (EHR) and Geographic Information System (GIS) data is a more robust method to identify and address health disparities in access to care. By harnessing the capabilities of hot and coldspot analysis and incorporating neighborhood-level characteristics, we can better understand the intricate interplay between social, economic, and environmental factors contributing to health disparities in access to care. This integrative approach can empower researchers and policymakers to develop targeted interventions and strategies to mitigate these disparities locally and promote equitable healthcare access. Considering that the San Francisco region is known for its high cost of living, examining the visual representation of ADI scores and disparities of access time to care can inform us about the potential interplay between social environment and barriers to accessing healthcare in ambulatory settings.

### Supplementary Information

**Additional file 1.**

## Data Availability

The dataset generated and/or analysed during the current study are not publicly available due to data owned and compiled by UCSF but are available from the corresponding author on reasonable request.

## Abbreviations

PT: Physical Therapy
EHR: Electronic Health Record
GIS: Geographic Information Systems
SES: Sociodemographics
ADI: Area Deprivation Index
CDW: Clinical Data Warehouse
PHDI: UCSF Population Health Data Initiatives
ACA: Affordable Care Act
