# Supplementary material for: Examining rehabilitation access disparities: an integrated analysis of electronic health record data and population characteristics through bivariate choropleth mapping
Source: BMC Health Serv Res. 2024 Feb 7;24:170. doi: 10.1186/s12913-024-10649-1 (PMC10848529; doi:10.1186/s12913-024-10649-1)
Supplement: Supplementary file 1 — Additional file 1. [file 12913_2024_10649_MOESM1_ESM.docx]

*Supplementary Material*

Sang S Pak, PT, DPT, Madeline Ratoza, PT, DPT, PhD(C), Victor Cheuy, PhD

**Data Preparation**

Original patient-level dataset containing three access metrics were extracted from CDW with linked with geocoded data prepared by the UCSF PHDI geocoding team.^1^

Publicly available Area Deprivation Index Score dataset (2020 version) was downloaded (<https://www.neighborhoodatlas.medicine.wisc.edu/> and matched to patient ZIP codes. Additional parameters include:

- - All ADI data are generated at the block group level
  - Downloaded 9-digitZIP code to match with patient ZIP codes
  - Geocoded patient addresses were assigned an ADI national rank according to their residential census block group. Of note, higher ADI rank indicates more disadvantage. Analyses used ADI rank quintiles.

Distance between clinic and place of residency was calculated using ArcGIS Pro’s Near Analysis function.^2^ This function calculates distance and additional proximity information between the input features (patient resident location) and the closest feature (clinic) in another layer or feature class.

**Geospatial clustering – Hot Spot and Cold Spot Analysis**

First step is to calculate the "fixed distance band"^2:^ features that are closer to each other within a critical distance receive a higher weight in spatial computation and so use of the fixed distance band is recommended for G_i_^∗^ statistics.^4^ The value for the fixed distance band was calculated by choosing the minimum neighbors required for analyzing spatial relationships to ensure the input feature had at least ten neighbors **(Supplemental Table 1)**

Second step is to determine the average distance to the Nth nearest feature **(Supplemental Table 2 & Supplemental Figure 1)**. For example, we calculated the average distance to the Nth nearest feature^2^ using ArcGIS Pro Near Analysis function with a feature of interest (i.e, referral metrics) to calculate the maximum distance between clusters, identify whether initial spatial patterns of clustering exist and calculate spatial statistics^5^

**Supplemental Table 1. Distance Band Summary**

| **Parameters for distance band** | **Distance in Meters** |
| --- | --- |
| Minimum 10 neighbor distance | 0.000000 |
| Average 10 neighbor distance | 152.70 |
| Maximum 10 neighbor distance | 1751.37 |

**Supplemental Table 2. Nth Nearest Feature Summary**

| **Parameters for Nth Nearest Feature** | **Results** |
| --- | --- |
| Observed Mean Distance (Meters) | 31.66 |
| Expected Mean Distance (Meters) | 57.56 |
| Nearest Neighbor Ratio | 0.550050 |
| z-score | -87.63 |
| p-value | P>.0001 |

**Supplemental Figure 1. Average Nearest Neighbor Summary**


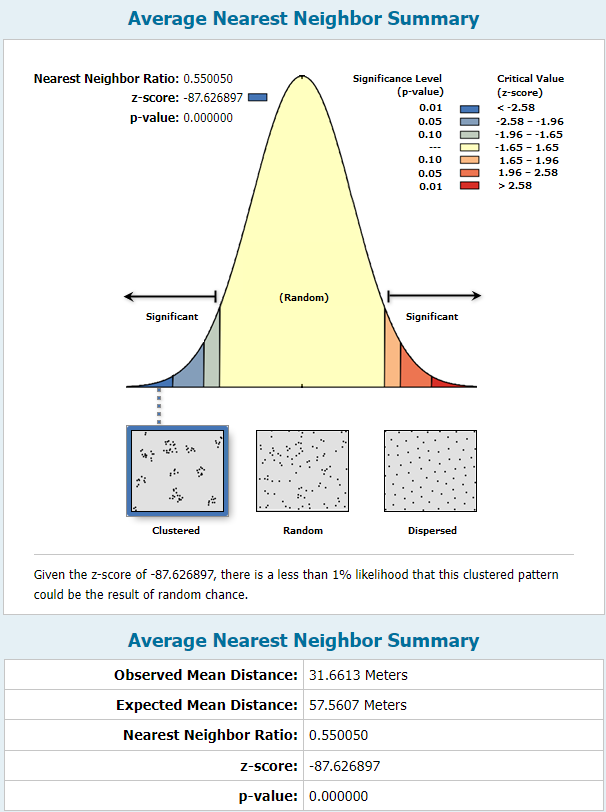


The third step is to calculate the critical distance or distance bandwidths at maximum clustering using Incremental spatial autocorrelation^6^ **(Supplemental Figure 2 & Supplemental Table 3)**. We used spatial autocorrelation at multiple distances and plotted the corresponding z-scores on a line graph **(Supplemental Figure 2)**. The z-scores indicate the level of spatial clustering intensity, and statistically significant z-scores signify the distances at which the maximum clustering is detected **(Supplemental Figure 3)**.

**Supplemental Figure 2. Spatial Autocorrelation with Calculated Peaks**


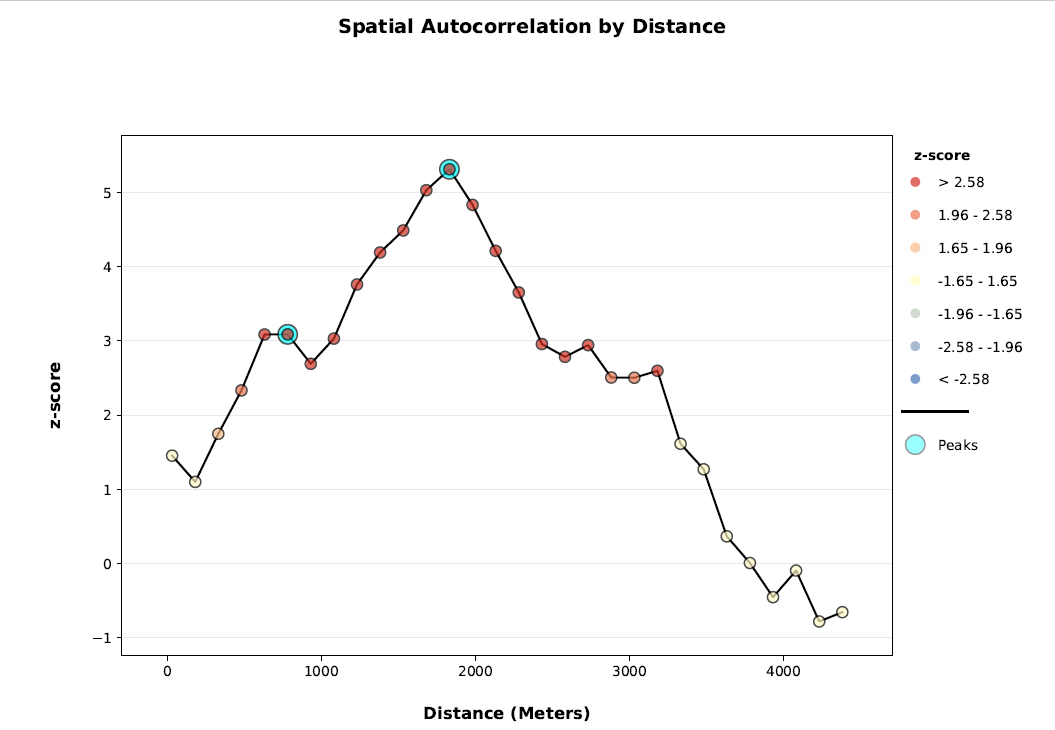


**Supplemental Table 3. Spatial Autocorrelation Global Moran’s I Table by Distance**


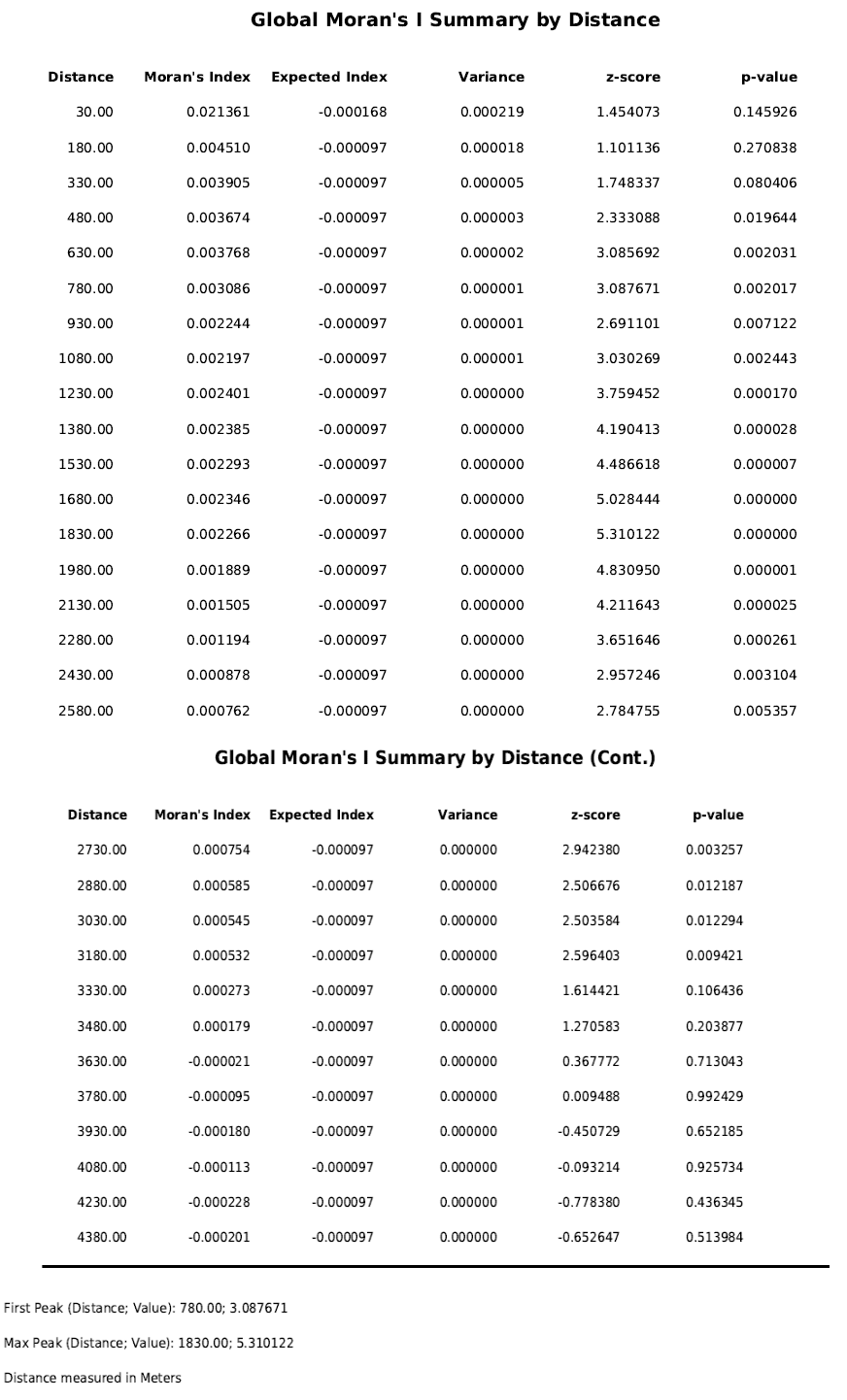


**Supplemental Figure 3. Example of Spatial Autocorrelation Report Demonstrating Clustered Detection**


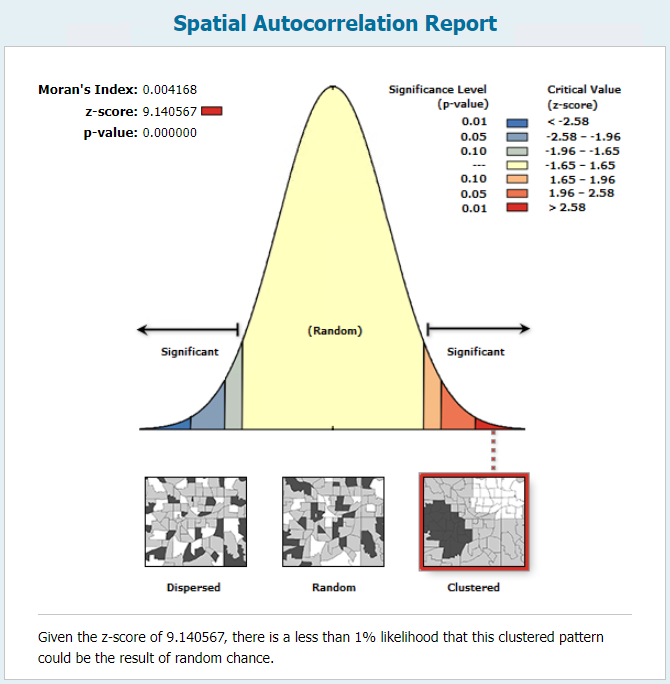


The final step was to proceed with Getis-Ord G_i_^∗^ statistic^7^ with hot spot (i.e. highest duration of access time) and cold spot (i.e. lowest duration of access time) analyses to test the statistical significance of local clusters and the spatial extent of these clusters (95% CI).^7^ GI statistics contain a z-score^7^ and clusters with a 95% significance level from a two-tailed normal distribution. A z-score close to zero and a p-value greater than 0.05 suggest complete spatial randomness within the study area. On the other hand, a positive z-score and a p-value less than 0.05 signify the clustering of high values. The hot and cold spot analysis results were calculated with a 95% CI. Lastly, clusters were compared using the False Discovery Rate correction in the analysis to account for potential false positive hotspots.^8^

References

1. Population Health Data Initiative | Population Health and Health Equity. https://pophealth.ucsf.edu/population-health-data-initiative.

2. Find the nearest feature—ArcGIS Pro | Documentation.

3. Modeling spatial relationships—ArcGIS Pro | Documentation. https://pro.arcgis.com/en/pro-app/2.8/tool-reference/spatial-statistics/modeling-spatial-relationships.htm.

4. Grekousis, G. *Spatial Analysis Methods and Practice: Describe – Explore – Explain through GIS*. (Cambridge University Press, 2020). doi:DOI: 10.1017/9781108614528.

5. Calculate Distance Band from Neighbor Count (Spatial Statistics)—ArcGIS Pro | Documentation. https://pro.arcgis.com/en/pro-app/latest/tool-reference/spatial-statistics/calculate-distance-band-from-neighbor-count.htm.

6. Peeters, A. *et al.* Getis–Ord\textquoterights hot- and cold-spot statistics as a basis for multivariate spatial clustering of orchard tree data. *Comput. Electron. Agric.* **111**, 140–150 (2015).

7. Getis, A. & Ord, J. K. The analysis of spatial association by use of distance statistics. *Geogr. Anal.* **24**, 189–206 (2010).

8. Tegegne, T. K., Chojenta, C., Getachew, T., Smith, R. & Loxton, D. Service environment link and false discovery rate correction: Methodological considerations in population and health facility surveys. *PLoS One* **14**, e0219860 (2019).
